# Frailty and outcomes in older adults hospitalized for epilepsy: a nationwide inpatient sample study

**DOI:** 10.1186/s12877-026-07790-3

**Published:** 2026-06-10

**Authors:** Yue-Loong Hsin, Min-Fei Chuang

**Affiliations:** 1 Department of Neurology, Chung Shan Medical University Hospital, 110, Section 1, Jianguo North Road, Taichung, 40201 Taiwan; 2 Department of Neurology, School of Medicine, Chung Shan Medical University, 110, Section 1, Jianguo North Road, Taichung, 40201 Taiwan

**Keywords:** Epilepsy, Frailty, In-hospital outcome, Complication, Johns Hopkins Adjusted Clinical Groups (ACG) frailty indicator, Nationwide Inpatient Sample (NIS)

## Abstract

**Background:**

Epilepsy has a bimodal incidence distribution, with a second peak in older age. Older adults with epilepsy represent a clinically vulnerable and relatively understudied population, and frailty may be an important determinant of outcomes in this setting. This study examined the prevalence of frailty among older adults hospitalized with epilepsy and its associations with in-hospital outcomes.

**Methods:**

This population-based retrospective study used data from the Nationwide Inpatient Sample (2005–2022). Hospitalized adults aged ≥ 60 years with a primary diagnosis of epilepsy were included. Frailty was defined using the Johns Hopkins Adjusted Clinical Groups frailty indicator. Propensity score matching was performed to balance baseline characteristics. Multivariable regression analyses were used to examine the associations between frailty and in-hospital outcomes, including major complications, prolonged length of stay (LOS), unfavorable discharge, total hospital costs, and in-hospital mortality.

**Results:**

Among 176,884 eligible hospitalizations, 57.7% were classified as frail. After matching, 140,094 patients were analyzed. Frailty was associated with higher risks of major complications (aOR 1.17, 95% CI 1.14–1.20), urinary tract infection (aOR 1.26, 95% CI 1.22–1.30), prolonged LOS (aOR 1.34, 95% CI 1.31–1.38), and unfavorable discharge (aOR 1.84, 95% CI 1.80–1.89), as well as increased total hospital costs. These associations were consistent across subgroups defined by age, intractable epilepsy status, and substance abuse.

In contrast, frailty was associated with a lower risk of in-hospital mortality (aOR 0.77, 95% CI 0.71–0.84).

**Conclusions:**

Frailty was highly prevalent among older adults hospitalized with epilepsy and is associated with worse in-hospital outcomes and greater healthcare resource utilization. The observed lower in-hospital mortality among frail patients likely reflects differences in discharge patterns and care pathways. These findings support the clinical relevance of frailty recognition in older adults hospitalized with epilepsy.

**Supplementary Information:**

The online version contains supplementary material available at 10.1186/s12877-026-07790-3.

## Introduction

In recent years, increasing clinical attention has been directed toward epilepsy and frailty in the aging population. Epilepsy has a bimodal incidence distribution, with peaks in early life and in older age, and older adults account for a substantial proportion of new-onset epilepsy cases [1–3]. A comprehensive United States (US) national survey revealed a substantial 24% surge in the incidence of epilepsy from 2010 to 2015, with a particularly noteworthy rise among people over 60 years of age [4]. Medicare data further underscores the significance of this issue, estimating the incidence and prevalence rates of epilepsy among older adults in the US at 2.41 cases/1,000 persons and 10.8 cases/1,000 persons, respectively [5].

Older adults with epilepsy represent a clinically vulnerable population. Aging-related neurologic disorders, including stroke, traumatic brain injury, and Alzheimer’s disease, contribute importantly to epilepsy in later life and add to its diagnostic and management complexity [6, 7].

In parallel, frailty is increasingly recognized as a critical determinant of health in older adults. Frailty is characterized by diminished physiological reserves, heightened vulnerability to stressors, and reduced resilience, and is now recognized as a critical factor in the health of older adults [8, 9]. The manifestations of frailty involve a decline across multiple physiological systems, predisposing individuals to an elevated risk of adverse health outcomes that includes falls, disability, hospitalizations, and mortality [10]. Frail individuals often encounter difficulties in recovering from illnesses or stressors, leading to a decline in functional capacity [11, 12]. Frailty has been shown to be prevalent among older individuals admitted to the hospital for acute, non-elective reasons, and it consistently serves as a predictive factor for mortality, length of hospital stay (LOS), and the likelihood of being discharged to a location other than home, and as the severity of frailty increases so does the risk of these adverse outcomes [13].

As the population ages, the intersection between epilepsy and frailty becomes increasingly pertinent [14]. Frailty is often overlooked in the context of epilepsy [15]. Although frailty has been linked to poor outcomes across a wide range of medical conditions, data specifically addressing its prevalence and clinical impact in older adults hospitalized with epilepsy remain limited. Therefore, this study aimed to determine the prevalence of frailty among older adults hospitalized with epilepsy and to evaluate its associations with in-hospital complications, length of stay, discharge outcomes, healthcare utilization, and in-hospital mortality using a nationally representative US database.

## Methods

### Data source

Data for this study were obtained from the Nationwide Inpatient Sample (NIS), the largest publicly available all-payer inpatient database in the US. The NIS, developed by the Healthcare Cost and Utilization Project (HCUP) and maintained by the Agency for Healthcare Research and Quality (AHRQ), contains a stratified 20% sample of inpatient discharges from participating hospitals across the country [16]. Each discharge record includes information on diagnoses, procedures, demographic characteristics, admission type, discharge disposition, and length of stay. Sampling weights supplied by HCUP were applied to generate nationally representative estimates in accordance with the NIS survey design. 

More details on the design and data framework of the NIS are available at: https://health.gov/healthypeople/objectives-and-data/data-sources-and-methods/data-sources/healthcare-cost-and-utilization-project-national-nationwide-inpatient-sample-hcup-nis.

### Study design and ethics considerations

This was a population-based retrospective study that analyzed in-patient data collected at patient discharge from 2005 to 2022 and maintained in the HCUP-NIS administrative database. All data were obtained from the Online HCUP Central Distributor (https://www.distributor.hcup-us.ahrq.gov/). As this study utilized data from the NIS database, direct involvement of patients and the public did not occur. Given that patient data within the NIS database are de-identified, the requirement for signed informed consent was waived.

### Study population

Hospitalized patients aged ≥ 60 years with a primary diagnosis of epilepsy in the NIS database from 2005 to 2022 were eligible for inclusion. The International Classification of Diseases, Ninth Revision and Tenth Revisions, Clinical Modification (ICD-9-CM and ICD-10-CM) diagnostic and procedure codes were utilized to identify admission diagnosis and procedures in the database. Patients hospitalized and diagnosed with ‘febrile seizure’, ‘post-traumatic seizures’, or ‘other convulsions’, traumatic brain injury (TBI), or central nervous system (CNS) tumor, were excluded, as were patients with missing data on sex, mortality status, LOS, total hospital costs, discharge destination, and sample weight. The ICD diagnostic and procedure codes used were listed in Supplementary Table 1. Patients were subsequently classified into two groups: frail and non-frail.

### Ascertainment of frailty

Frailty status was determined using the Johns Hopkins Adjusted Clinical Groups (ACG) frailty indicator, a validated tool designed for use with administrative claims data [17]. The ACG frailty measure assigns a binary classification based on the presence of specific diagnosis clusters identified through ICD-9-CM and ICD-10-CM codes [18, 19]. These clusters include conditions such as malnutrition, dementia, impaired vision, pressure ulcers, urinary or fecal incontinence, weight loss, socioeconomic barriers, and mobility limitations.

Patients were classified as frail if they met at least one of these criteria. Details of the coding algorithm are provided in Supplementary Table 2.

### Main outcomes and study variables

The outcomes of interest were: (1) major complications, including acute myocardial infarction (AMI) and other cardiac complications, cerebrovascular accident (CVA), venous thromboembolism (VTE), pneumonia, urinary tract infection (UTI), and sepsis / systemic inflammatory response syndrome (SIRS); (2) prolonged LOS, defined as having a LOS ≥ 75th percentile among the study population; (3) unfavorable discharge (defined as discharged to a long-term care facility); (4) total hospital costs, and (5) in-hospital mortality. The ICD codes for defining the complications are documented in Supplementary Table 1.

Data extracted from the NIS database included patient demographics (age, sex, race/ethnicity), insurance information (primary payer), household income quartile—as estimated from ZIP code income data updated annually—smoking status, CNS infection, Parkinson disease; ischemic stroke, transient ischemic attack (TIA), hemorrhagic stroke, epilepsy characteristics (intractability), and whether or not received epilepsy surgery during admission. These conditions were identified using corresponding ICD diagnostic and procedure codes (Supplementary Table S1).

Elixhauser comorbidity scores, year of hospital admission, admission emergent status, and whether admission occurred on a weekend were also included as covariates. Household income quartiles were obtained from the NIS, and estimated from the household income of residents in the same ZIP code as the patient (https://hcup-us.ahrq.gov/db/vars/zipinc_qrtl/nisnote.jsp). Since these estimates are updated annually, the value range categories vary by year. Hospital-level data extracted included hospital bed size, region, and hospital location/teaching status.

### Statistical analysis

Descriptive statistics were used to summarize patient demographics and clinical characteristics, with categorical variables presented as counts and weighted percentages and continuous variables as means with standard errors (SE). Group comparisons for categorical variables were conducted using the Rao-Scott chi-square test, while weighted mean differences for continuous variables were analyzed using survey methods that account for stratification, clustering, and sampling weights, ensuring valid and robust statistical inferences within the context of complex survey designs.

To minimize confounding, propensity score matching (PSM) was performed using a 1:1 ratio, with age, sex, race, insurance status / primary payer, Elixhauser comorbidity score, and year of admission as the matching variables. The matching process followed a one-to-many approach [20]. The method prioritizes “best” matches first and then proceeds with “next-best” matches until no more can be made. Logistic and linear regression analyses were used to calculate odds ratios (ORs), estimated coefficients (Betas) and 95% confidence intervals (CIs) for binary outcomes and total hospital cost, respectively. The multivariable models were adjusted for covariates, with an imbalance still between the matched groups, including metastatic cancer. Standardized mean differences (SMDs) ≥ 0.1 are shown in bold. A two-sided p-value of < 0.05 was considered statistically significant. All analyses accounted for the NIS’s complex survey design to ensure accurate national estimates. Statistical analyses were conducted using SAS version 9.4 (SAS Institute Inc., Cary, NC, USA).

## Results

### Patient selection

**Fig. 1:**
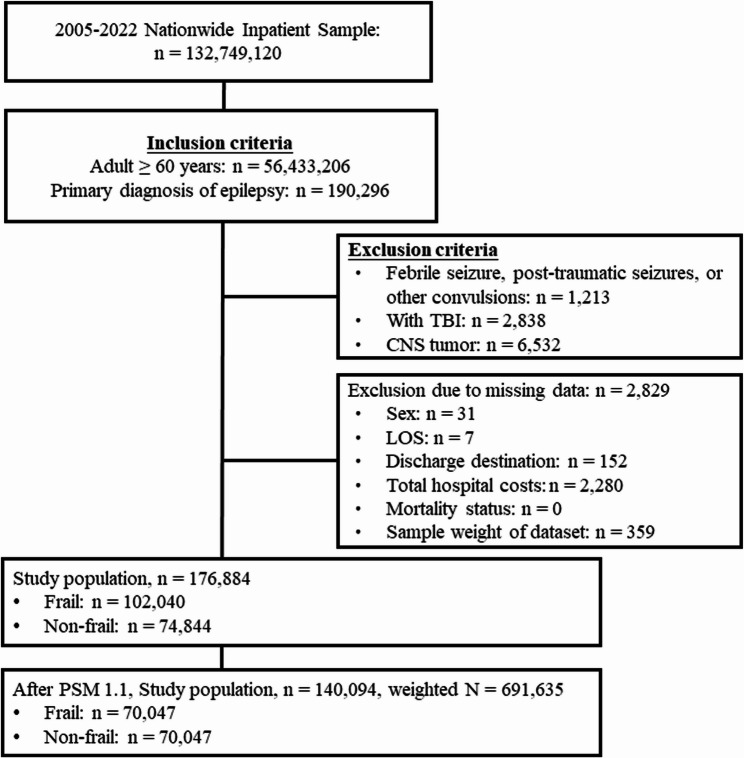
Flow diagram of patient selection

A flow diagram of the patient selection process is shown in Fig. 1. A total of 190,296 adults with epilepsy and > = 60 years from the NIS database from 2005 to 2022 were included. Patients with febrile seizures, post-traumatic seizures, or other convulsions (*n* = 1,213), TBI (*n* = 2,838), CNS tumor (*n* = 6,532), and those with missing data on sex, in-hospital outcomes, or weight values (*n* = 2,829) in the dataset were excluded. Finally, 176,884 patients were included in the study. Among all patients, 57.7% (102,040/176,884) were classified as frail.

After PSM, 140,094 patients remained for subsequent analyses, consisting of 70,047 frail patients and 70,047 non-frail patients. This sample represents a total of 691,635 hospitalizations in the entire US after weighting (Fig. 1).

### Characteristics of epilepsy patients categorized by frailty status

Statistics of the study outcomes, demographics, comorbidities, and hospital-related information for the study population are summarized in Supplementary Tables 3 and 4, while the corresponding statistics after PSM are presented in Tables 1 and 2.

**Table 1:** Characteristics of epilepsy patients categorized by frailty status after PSM

		Frailty		
	All patients (*n* = 140,094)	Frail (*n* = 70,047)	Non-frail (*n* = 70,047)	SMD
**Demography**				
Age, years	71.3 ± 0.03	71.3 ± 0.04	71.3 ± 0.04	< 0.001
60–69	68,761 (49.1)	34,283 (49.0)	34,478 (49.3)	0.008
70–79	44,908 (32.0)	22,425 (32.0)	22,483 (32.1)	
>= 80	26,425 (18.8)	13,339 (19.0)	13,086 (18.6)	
Sex				0.002
Male	71,710 (51.2)	35,878 (51.3)	35,832 (51.2)	
Female	68,384 (48.8)	34,169 (48.7)	34,215 (48.8)	
Race/ethnicity				0.017
White	81,737 (58.4)	41,152 (58.8)	40,585 (58.0)	
Black	32,168 (23.0)	15,921 (22.8)	16,247 (23.3)	
Hispanic	10,696 (7.6)	5282 (7.5)	5414 (7.7)	
Other/unknown	15,493 (10.9)	7692 (10.9)	7801 (11.0)	
Insurance status / primary payer				0.008
Medicare/Medicaid	118,081 (84.4)	59,145 (84.5)	58,936 (84.2)	
Private including HMO	16,454 (11.8)	8162 (11.7)	8292 (11.8)	
Self-pay/no-charge/other	5390 (3.9)	2659 (3.8)	2731 (3.9)	
Missing	169	81	88	
Household income				0.022
Q1	45,181 (33.0)	22,337 (32.6)	22,844 (33.4)	
Q2	34,279 (25.0)	17,127 (24.9)	17,152 (25.0)	
Q3	30,830 (22.4)	15,719 (22.8)	15,111 (22.0)	
Q4	26,873 (19.6)	13,456 (19.6)	13,417 (19.6)	
Missing	2931	1408	1523	
Smoking	45,011 (32.2)	22,398 (32.0)	22,613 (32.3)	0.006
CNS infection	691 (0.5)	319 (0.5)	372 (0.5)	0.010
Parkinson disease	1940 (1.4)	1212 (1.7)	728 (1.0)	0.058
Ischemic stroke	2655 (1.9)	1203 (1.7)	1452 (2.1)	0.026
TIA	1795 (1.3)	804 (1.1)	991 (1.4)	0.024
Hemorrhagic stroke	1768 (1.3)	800 (1.1)	968 (1.4)	0.022
Intractable epilepsy	7903 (5.7)	4022 (5.8)	3881 (5.6)	0.009
Epilepsy surgery	211 (0.2)	87 (0.1)	124 (0.2)	0.013
Elixhauser Comorbidities				
AIDS	371 (0.3)	213 (0.3)	158 (0.2)	0.015
Alcohol abuse	13,005 (9.3)	6583 (9.4)	6422 (9.2)	0.008
Anemia (chronic blood loss)	433 (0.3)	230 (0.3)	203 (0.3)	0.007
Anemia (deficiency)	15,171 (10.7)	8090 (11.5)	7081 (10.0)	0.046
Rheumatoid arthritis/collagen vascular diseases	3341 (2.4)	1775 (2.5)	1566 (2.2)	0.020
Congestive heart failure	19,075 (13.6)	9213 (13.2)	9862 (14.1)	0.027
Chronic pulmonary disease	26,919 (19.2)	14,190 (20.3)	12,729 (18.2)	0.053
Coagulopathy	4112 (2.9)	1977 (2.8)	2135 (3.0)	0.013
Diabetes (uncomplicated)	27,882 (19.9)	13,380 (19.1)	14,502 (20.6)	0.039
Diabetes (complicated)	15,321 (11.0)	7641 (11.0)	7680 (11.0)	0.002
Drug abuse	6132 (4.4)	3364 (4.8)	2768 (4.0)	0.041
Hypertension	101,905 (72.8)	50,986 (72.8)	50,919 (72.8)	0.002
Hypothyroidism	21,817 (15.6)	11,705 (16.7)	10,112 (14.4)	0.062
Liver disease	5057 (3.6)	2523 (3.6)	2534 (3.6)	< 0.001
Lymphoma	1151 (0.8)	470 (0.7)	681 (1.0)	0.033
Fluid/electrolyte disorders	50,222 (35.9)	25,748 (36.8)	24,474 (35.0)	0.038
Metastatic cancer	4443 (3.2)	1512 (2.2)	2931 (4.2)	**0.116**
Obesity	10,361 (7.4)	5085 (7.3)	5276 (7.6)	0.010
Peripheral vascular disorders	8598 (6.1)	4358 (6.2)	4240 (6.1)	0.007
Pulmonary circulation disorders	2884 (2.1)	1338 (1.9)	1546 (2.2)	0.020
Renal failure	27,673 (19.8)	13,504 (19.4)	14,169 (20.3)	0.024
Non-metastatic cancer	4286 (3.1)	1713 (2.5)	2573 (3.7)	0.071
Valvular disease	7510 (5.4)	3647 (5.2)	3863 (5.5)	0.013
Elixhauser comorbidity score				0.014
0	10,141 (7.2)	5022 (7.1)	5119 (7.3)	
1	25,679 (18.3)	12,690 (18.1)	12,989 (18.5)	
2	33,918 (24.2)	16,943 (24.2)	16,975 (24.2)	
3+	70,356 (50.3)	35,392 (50.6)	34,964 (50.0)	
Year of admission				0.008
2005–2009	23,592 (16.3)	11,753 (16.3)	11,839 (16.4)	
2010–2014	42,246 (30.0)	21,037 (29.9)	21,209 (30.1)	
2015–2018	37,260 (26.9)	18,752 (27.1)	18,508 (26.8)	
2019–2022	36,996 (26.7)	18,505 (26.7)	18,491 (26.7)	
Emergent admission	130,990 (93.7)	65,731 (94.0)	65,259 (93.3)	0.029
Missing	239	136	103	
Weekend admission	36,391 (26.0)	18,223 (26.0)	18,168 (26.0)	0.002
Hospital bed size				0.029
Small	21,295 (15.1)	11,009 (15.6)	10,286 (14.6)	
Medium	38,512 (27.6)	19,277 (27.6)	19,235 (27.6)	
Large	79,969 (57.3)	39,606 (56.7)	40,363 (57.8)	
Missing	318	155	163	
Hospital region				0.046
Northeast	31,767 (22.7)	15,597 (22.3)	16,170 (23.1)	
Midwest	29,444 (21.0)	15,275 (21.8)	14,169 (20.2)	
South	55,763 (39.8)	27,975 (39.9)	27,788 (39.7)	
West	23,120 (16.4)	11,200 (15.9)	11,920 (16.9)	
Hospital location/teaching status				0.010
Rural	11,774 (8.4)	5831 (8.3)	5943 (8.5)	
Urban nonteaching	40,273 (28.7)	20,270 (28.9)	20,003 (28.5)	
Urban teaching	87,729 (62.9)	43,791 (62.8)	43,938 (63.1)	
Missing	318	155	163	

Continuous variables are presented as mean ± SE; categorical variables are presented as unweighted counts (weighted percentage)

*Abbreviations*: *HMO* Health maintenance organization, *CNS* Central nervous system, *TIA* Transient ischemic attack, *AIDS* Acquired immunodeficiency syndrome, *PSM* Propensity score matching, *SMD* Standardized mean difference

SMDs ≥ 0.1 are shown in bold

**Table 2:** In-hospital outcomes of epilepsy patients categorized by frailty status after PSM

		Frailty		
	All patients (*n* = 140,094)	Frail (*n* = 70,047)	Non-frail (*n* = 70,047)	*p*-value
In-hospital outcomes				
Major complication	30,973 (22.1)	16,399 (23.4)	14,574 (20.8)	**< 0.001**
AMI	1697 (1.2)	811 (1.2)	886 (1.3)	0.064
VTE	2783 (2.0)	1366 (2.0)	1417 (2.0)	0.333
Pneumonia	6017 (4.3)	2954 (4.2)	3063 (4.4)	0.168
UTI	20,900 (14.9)	11,501 (16.4)	9399 (13.4)	**< 0.001**
Sepsis/SIRS	4411 (3.2)	2167 (3.1)	2244 (3.2)	0.235
Prolonged LOS, day ^a, b^	35,395 (25.8)	19,625 (28.5)	15,770 (23.0)	**< 0.001**
Unfavorable discharge ^a^	43,499 (31.6)	26,291 (38.1)	17,208 (25.1)	**< 0.001**
Total hospital cost, thousand USD	47.5 ± 0.4	47.9 ± 0.4	47.1 ± 0.4	0.064
In-hospital mortality	2565 (1.8)	1096 (1.6)	1469 (2.1)	**< 0.001**

Continuous variables are presented as mean ± SE; categorical variables are presented as unweighted counts (weighted percentage)

*Abbreviations*: *AMI* Acute myocardial infarction, *VTE* Venous thromboembolism, *UTI* Urinary tract infection, *SIRS* Systemic inflammatory response syndrome, *LOS* Length of hospital stay, *PSM* propensity score matching

^a^ Excluding patients who died in the hospital

^b^ Length of hospital stay ≥ 75th percentile is 6 days

*p*-values < 0.05 shown in bold

The mean age of all patients was 72.4 years. 53.0% were female, 59.8% were white, and 84.9% had insurance covered by Medicare/Medicaid. The most common comorbidity was hypertension (73.7%). 50.7% of patients had an Elixhauser comorbidity score > = 3.

Significant differences were observed in age, sex, race, insurance status / primary payer, hypothyroidism, metastatic cancer, Elixhauser comorbidity score, and year of admission between the two groups (Supplementary Table 3). And we also found that any major complication, AMI, UTI, prolonged LOS, unfavorable discharge, total hospital cost, and in-hospital mortality had significant differences between the two groups (Supplementary Table 4).

After PSM, the distribution of covariates was balanced between the groups, except for metastatic cancer (Table 1). For in-hospital outcomes, significant differences remained in any major complication, UTI, prolonged LOS, unfavorable discharge, and in-hospital mortality (Table 2).

### Association between frailty and outcomes in patients hospitalized for epilepsy

Table 3 shows the associations between frailty and outcomes in patients hospitalized for epilepsy. After adjustment in the multivariable analysis, frailty was significantly associated with higher risk of major complications (adjusted OR [aOR] = 1.17, 95% CI: 1.14–1.20, *p* < 0.001), prolonged LOS (aOR = 1.34, 95% CI: 1.31–1.38, *p* < 0.001), and unfavorable discharge (aOR = 1.84, 95% CI: 1.80–1.89, *p* < 0.001). Among major complications, compared with non-frail patients, frail patients had a significantly higher risk for UTI (aOR = 1.26, 95% CI: 1.22–1.30, *p* < 0.001). Frailty had significantly higher total hospital cost than those non-frail (aBeta = 0.93, 95% CI: 0.05–1.80, *p* = 0.039).

**Table 3:** Associations between frailty status and in-hospital outcomes in epilepsy patients

Outcomes		Univariate			Multivariable	
	Frailty	OR/Beta ^a^ (95% CI)	*p*-value		aOR / aBeta ^a, b^ (95% CI)	*p*-value
Major complication	Frail vs. Non-frail	1.16 (1.13, 1.19)	**< 0.001**		1.17 (1.14, 1.20)	**< 0.001**
AMI	Frail vs. Non-frail	0.91 (0.83, 1.01)	0.064		0.91 (0.83, 1.00)	0.053
VTE	Frail vs. Non-frail	0.96 (0.89, 1.04)	0.334		1.00 (0.92, 1.07)	0.901
Pneumonia	Frail vs. Non-frail	0.96 (0.91, 1.02)	0.168		0.97 (0.92, 1.02)	0.269
UTI	Frail vs. Non-frail	1.27 (1.23, 1.31)	**< 0.001**		1.26 (1.22, 1.30)	**< 0.001**
Sepsis/SIRS	Frail vs. Non-frail	0.96 (0.91, 1.02)	0.235		0.97 (0.91, 1.03)	0.344
Prolonged LOS, day ^c, d^	Frail vs. Non-frail	1.33 (1.30, 1.37)	**< 0.001**		1.34 (1.31, 1.38)	**< 0.001**
Unfavorable discharge ^c^	Frail vs. Non-frail	1.84 (1.79, 1.88)	**< 0.001**		1.84 (1.80, 1.89)	**< 0.001**
Total hospital cost, thousand USD	Frail vs. Non-frail	0.83 (-0.05, 1.70)	0.064		0.93 (0.05, 1.80)	**0.039**
In-hospital mortality	Frail vs. Non-frail	0.74 (0.68, 0.81)	**< 0.001**		0.77 (0.71, 0.84)	**< 0.001**

*Abbreviations*: *AMI* Acute myocardial infarction, *VTE* Venous thromboembolism, *UTI* Urinary tract infection, *SIRS* Systemic inflammatory response syndrome, *LOS* Length of hospital stay, *OR* Odds ratio, *aOR* Adjusted odds ratio, *CI* Confidence interval

^a^ Beta coefficients represent the effects of independent variables on the predicted outcomes of total hospital cost

^b^ All multivariable models adjusted for all variables that are not balanced (SMD ≥ 0.1) after PSM, including metastatic cancer

^c^ Excluding patients who died in the hospital

^d^ Length of hospital stay ≥ 75th percentile is 6 days

*p*-value < 0.05 shown in bold

In contrast, frailty was significantly associated with lower risk of in-hospital mortality (aOR = 0.77, 95% CI: 0.71–0.84, *p* < 0.001) **(**Table 3**)**.

### Stratified associations between frailty and outcomes in patients hospitalized for epilepsy

Results of the stratified analyses by age, intractable epilepsy status, and substance abuse are shown in Table 4. Among all subgroups, frail patients had a consistently and significantly increased risk for any major complication, UTI, prolonged LOS, and unfavorable discharge, except for the risk of any major complication in the subgroup of patients with substance abuse. And frail patients had a consistently and significantly increased total hospital cost in the subgroups of age < 70 years, patients without intractable epilepsy, or patients with substance abuse.

**Table 4:** Stratified associations between frailty and in-hospital outcomes in epilepsy patients

Outcomes		Multivariable	
	Frailty	aOR / aBeta ^a, b^ (95% CI)	*p*-value
Age < 70 years (*n* = 68,761)			
Major complication	Frail vs. Non-frail	1.14 (1.09, 1.18)	**< 0.001**
AMI	Frail vs. Non-frail	0.90 (0.79, 1.04)	0.151
VTE	Frail vs. Non-frail	0.95 (0.85, 1.07)	0.412
Pneumonia	Frail vs. Non-frail	0.95 (0.87, 1.02)	0.162
UTI	Frail vs. Non-frail	1.25 (1.20, 1.31)	**< 0.001**
Sepsis/SIRS	Frail vs. Non-frail	0.99 (0.91, 1.08)	0.875
Prolonged LOS, day ^c, d^	Frail vs. Non-frail	1.38 (1.33, 1.43)	**< 0.001**
Unfavorable discharge ^c^	Frail vs. Non-frail	1.77 (1.71, 1.84)	**< 0.001**
Total hospital cost, thousand USD	Frail vs. Non-frail	0.89 (0.03, 1.75)	**0.042**
In-hospital mortality	Frail vs. Non-frail	0.75 (0.65, 0.86)	**< 0.001**
Age 70 + years (*n* = 71,333)			
Major complication	Frail vs. Non-frail	1.19 (1.15, 1.23)	**< 0.001**
AMI	Frail vs. Non-frail	0.92 (0.80, 1.05)	0.190
VTE	Frail vs. Non-frail	1.03 (0.93, 1.15)	0.524
Pneumonia	Frail vs. Non-frail	0.99 (0.92, 1.07)	0.810
UTI	Frail vs. Non-frail	1.27 (1.22, 1.32)	**< 0.001**
Sepsis/SIRS	Frail vs. Non-frail	0.95 (0.87, 1.03)	0.228
Prolonged LOS, day ^c, d^	Frail vs. Non-frail	1.31 (1.27, 1.36)	**< 0.001**
Unfavorable discharge ^c^	Frail vs. Non-frail	1.92 (1.86, 1.99)	**< 0.001**
Total hospital cost, thousand USD	Frail vs. Non-frail	0.69 (-5.13, 6.51)	0.817
In-hospital mortality	Frail vs. Non-frail	0.78 (0.71, 0.86)	**< 0.001**
Intractable epilepsy (No) (*n* = 132,191)			
Major complication	Frail vs. Non-frail	1.17 (1.14, 1.20)	**< 0.001**
AMI	Frail vs. Non-frail	0.92 (0.83, 1.02)	0.094
VTE	Frail vs. Non-frail	1.01 (0.93, 1.09)	0.799
Pneumonia	Frail vs. Non-frail	0.97 (0.91, 1.02)	0.207
UTI	Frail vs. Non-frail	1.26 (1.22, 1.30)	**< 0.001**
Sepsis/SIRS	Frail vs. Non-frail	0.97 (0.91, 1.03)	0.320
Prolonged LOS, day ^c, d^	Frail vs. Non-frail	1.35 (1.31, 1.38)	**< 0.001**
Unfavorable discharge ^c^	Frail vs. Non-frail	1.85 (1.81, 1.90)	**< 0.001**
Total hospital cost, thousand USD	Frail vs. Non-frail	0.89 (0.03, 1.75)	**0.042**
In-hospital mortality	Frail vs. Non-frail	0.78 (0.72, 0.85)	**< 0.001**
Intractable epilepsy (Yes) (*n* = 7,903)			
Major complication	Frail vs. Non-frail	1.13 (1.01, 1.26)	**0.033**
AMI	Frail vs. Non-frail	0.75 (0.50, 1.13)	0.171
VTE	Frail vs. Non-frail	0.83 (0.63, 1.08)	0.168
Pneumonia	Frail vs. Non-frail	1.05 (0.85, 1.30)	0.660
UTI	Frail vs. Non-frail	1.27 (1.11, 1.44)	**< 0.001**
Sepsis/SIRS	Frail vs. Non-frail	0.99 (0.80, 1.22)	0.900
Prolonged LOS, day ^c, d^	Frail vs. Non-frail	1.27 (1.15, 1.40)	**< 0.001**
Unfavorable discharge ^c^	Frail vs. Non-frail	1.69 (1.53, 1.87)	**< 0.001**
Total hospital cost, thousand USD	Frail vs. Non-frail	0.69 (-5.13, 6.51)	0.817
In-hospital mortality	Frail vs. Non-frail	0.62 (0.48, 0.81)	**< 0.001**
Substance abuse (No) (*n* = 122,935)			
Major complication	Frail vs. Non-frail	1.18 (1.15, 1.21)	**< 0.001**
AMI	Frail vs. Non-frail	0.91 (0.82, 1.02)	0.090
VTE	Frail vs. Non-frail	0.99 (0.91, 1.08)	0.836
Pneumonia	Frail vs. Non-frail	0.97 (0.92, 1.03)	0.341
UTI	Frail vs. Non-frail	1.27 (1.23, 1.31)	**< 0.001**
Sepsis/SIRS	Frail vs. Non-frail	0.98 (0.92, 1.05)	0.576
Prolonged LOS, day ^c, d^	Frail vs. Non-frail	1.33 (1.30, 1.37)	**< 0.001**
Unfavorable discharge ^c^	Frail vs. Non-frail	1.86 (1.82, 1.91)	**< 0.001**
Total hospital cost, thousand USD	Frail vs. Non-frail	0.48 (-0.45, 1.41)	0.313
In-hospital mortality	Frail vs. Non-frail	0.77 (0.71, 0.84)	**< 0.001**
Substance abuse (Yes) (*n* = 17,159)			
Major complication	Frail vs. Non-frail	1.06 (0.98, 1.15)	0.120
AMI	Frail vs. Non-frail	0.88 (0.70, 1.10)	0.265
Cardiogenic shock	Frail vs. Non-frail	0.76 (0.30, 1.91)	0.561
VTE	Frail vs. Non-frail	1.03 (0.82, 1.29)	0.793
Pneumonia	Frail vs. Non-frail	0.95 (0.82, 1.11)	0.531
UTI	Frail vs. Non-frail	1.19 (1.08, 1.33)	**< 0.001**
Sepsis/SIRS	Frail vs. Non-frail	0.90 (0.77, 1.05)	0.161
Prolonged LOS, day ^c, d^	Frail vs. Non-frail	1.39 (1.30, 1.48)	**< 0.001**
Unfavorable discharge ^c^	Frail vs. Non-frail	1.74 (1.62, 1.87)	**< 0.001**
Total hospital cost, thousand USD	Frail vs. Non-frail	3.66 (1.28, 6.04)	**0.003**
In-hospital mortality	Frail vs. Non-frail	0.77 (0.59, 1.00)	0.053

*Abbreviations*: *AMI* Acute myocardial infarction, *VTE* Venous thromboembolism, *UTI* Urinary tract infection, *SIRS* Systemic inflammatory response syndrome, *LOS* Length of hospital stay, *aOR* Adjusted odds ratio, *CI* Confidence interval, *SMD* Standardized mean difference

^a^ Beta coefficients represent the effects of independent variables on the predicted outcomes of total hospital cost

^b^ All multivariable models adjusted for all variables that are not balanced (SMD ≥ 0.1) after PSM, including metastatic cancer

^c^ Excluding patients who died in the hospital

^d^ Length of hospital stay ≥ 75th percentile is 6 days

*p*-value < 0.05 shown in bold

In-hospital mortality was decreased risk among all subgroups, except for the subgroup of patients with substance abuse **(**Table 4**)**.

## Discussion

In this comprehensive analysis using data from the NIS, we found Among older adults hospitalized with epilepsy, 58% were classified as frail. Further, frailty was independently associated with a higher risk of adverse in-hospital outcomes, including major complications, prolonged length of stay, and unfavorable discharge. Frail patients also had a higher risk of urinary tract infection and incurred greater total hospital costs. Stratified analyses demonstrated that these associations were largely consistent across subgroups defined by age, intractable epilepsy status, and substance abuse. Notably, frailty was associated with a lower risk of in-hospital mortality, a finding that warrants cautious interpretation. Overall, these findings suggest that frailty is an important clinical factor associated with increased morbidity and healthcare resource utilization among older adults hospitalized for epilepsy, underscoring the need for targeted management strategies in this vulnerable population.

The incidence of epilepsy increases with age, as does the frequency of frailty, and with the aging population both are more commonly seen [13, 14]. Both frailty and epilepsy are associated with worse medical and surgical outcomes, and this is especially notable in older patients [13, 14]. In addition, with the aging population, multimorbidity, multiple health issues in a single person, is becoming more common and persons with epilepsy and multiple other health conditions are at increased risk of depression and suicide, premature death, lower quality of life, and require more hospital admissions and have higher health care costs [21].

Although the exact incidence and prevalence in older adults are hard to determine, but it is known that the incidence of epilepsy increases markedly after age 60, and persons older than 60 years are also at increased risk of acute symptomatic seizures [22, 23]. Researchers have also commented that in addition to chronological age, factors such as frailty status needed to be taken into account in the evaluation and treatment of older individuals with epilepsy [22].

Patients with epilepsy generally respond well to the variety of antiseizure medications (ASMs) currently available, with nearly 70% effectively managed in outpatient settings.

However, hospitalization is often required for patients experiencing severe or frequent seizures. The main reasons for hospital admission include new-onset seizures, status epilepticus, and epilepsy surgery [24, 25]. Although numerous studies have highlighted the prognostic significance of frailty in hospitalizations for a range of conditions, reports specifically addressing frailty as a prognostic factor for epilepsy-related hospitalizations remain limited [26–28]. Estes et al. [29] examined 696 patients who received epilepsy surgery from 2015 to 2019. The results showed that frailty status as defined using the 5-item modified frailty index (mFI-5), and not patient age, was significantly predictive of prolonged LOS and discharge to a facility other than home. Subota et al. [30] examined risk factors for dementia development, frailty, and mortality in 1,048 older adults with epilepsy. The results showed that Charlson comorbidity score and sleep disturbances at the time of epilepsy diagnosis were associated with increased risk of developing dementia, where baseline dementia and baseline frailty were significantly associated with increased risk of death. These findings appear to indicate that frailty is a significantly stronger prognostic factor than chronological age in predicting outcomes for epilepsy and its associated surgical treatments.

In alignment with previous findings, the current study corroborates that frailty is significantly associated with adverse outcomes of epilepsy admissions, transcending age along with other demographic and clinical variables.

In our analysis, 58% of older adults hospitalized with epilepsy were classified as frail, representing a substantial burden in this population. Several mechanisms may contribute to the high prevalence of frailty among patients hospitalized with epilepsy. First, epilepsy in older adults is frequently associated with underlying neurologic conditions, such as prior stroke or neurodegenerative disease, which are themselves closely linked to frailty. Second, recurrent seizures and their sequelae—including falls, injuries, and functional decline—may accelerate physical vulnerability and contribute to frailty progression. Third, the use of antiseizure medications, particularly in older individuals, may lead to adverse effects such as sedation, cognitive impairment, and reduced mobility, further increasing frailty risk [31].

Finally, shared pathophysiological mechanisms, including chronic inflammation, oxidative stress, and mitochondrial dysfunction, may contribute to both epilepsy and frailty [32, 33].

In our analyses, frailty was consistently associated with worse in-hospital outcomes among older adults hospitalized with epilepsy, including higher risks of UTI, prolonged stay, and unfavorable discharge. These findings are biologically and clinically plausible, as frailty reflects reduced physiological reserve and impaired stress response, predisposing patients to complications during acute illness [34]. In the context of epilepsy, recurrent seizures, immobility, and comorbid neurologic conditions may further increase vulnerability to infections and functional decline. Frail patients may also require more intensive care and longer recovery, contributing to greater healthcare utilization. The higher rate of unfavorable discharge, including transfer to post-acute care facilities, further highlights the substantial functional impact of frailty in this population.

In our analysis, frailty was associated with a lower risk of in-hospital mortality, a finding that appears counterintuitive and should be interpreted with caution. This result should not be interpreted as indicating a protective effect of frailty. Rather, one possible explanation is that frail patients may follow different care-transition pathways after acute medical stabilization.

Because frail patients are more likely to require post-acute or long-term institutional care, discharge planning and transfer to such facilities may be initiated earlier once they are considered stable for transfer. Consequently, these patients may leave the acute-care hospital before subsequent deterioration occurs. Since the NIS captures only deaths occurring during the index hospitalization and does not provide post-discharge follow-up, any later deterioration or death after discharge would not be counted as in-hospital mortality. Thus, the observed lower in-hospital mortality does not necessarily imply a lower overall mortality risk among frail patients. Instead, it may indicate that part of the mortality burden among frail patients occurs after transfer out of the acute-care hospital and is therefore not captured by the NIS. However, this remains a hypothesis rather than a mechanism that can be directly confirmed using the NIS dataset.

Quality of life is important to address in older persons, especially those with many comorbid conditions, including frailty and epilepsy. In an interesting study, Pervin et al. [35] studied quality of life in cognitively normal geriatric patients with epilepsy. The study included 927 community dwelling older persons, and found that incident epilepsy, irrespective of seizure control, impaired quality of life. Another recent study examined frailty in older adults with epilepsy, and found that frailty was significantly associated with decreased toleration of anti-seizure medications [36]. The current study did not evaluate quality of life, as it relied on a hospital-based database that also lacks information on medication usage. This gap underscores the need for future research to include these variables, enhancing our comprehension of how frailty interacts with these factors to influence outcomes.

### Clinical implications

Our findings highlight the importance of developing strategies specifically targeted at frailty in older patients with epilepsy, to mitigate its adverse impacts. A recent study documented that, not specific for epilepsy, participation in a multidomain intervention program during hospitalization not only improved the functional status of frail older individuals but also decreased the length of stays, medical costs, and readmission rates, underscoring the potential benefits of integrating frailty-focused care models into the management of older adults hospitalized with epilepsy. In summary, the evidence suggests that frailty assessments and tailored interventions for older individuals with epilepsy are recommended to enhance healthcare efficiency and reduce system burden [37].

### Strengths and limitations

The primary strength of the present study lies in its utilization of a large and representative national database, reflective of the entire US national population. Our analysis examined the influence of frailty on patients hospitalized for epilepsy, with careful adjustment for factors including patient demographic characteristics, comorbidities, and hospital features.

Nevertheless, several limitations should be acknowledged. First, the use of ICD coding systems to define epilepsy, comorbidities, and complications introduces the potential for coding errors and misclassification. In particular, the identification of intractable epilepsy based on diagnosis codes may be insensitive and subject to under-coding, and detailed measures of epilepsy severity—such as seizure frequency and disease duration—were not available in the NIS database. Therefore, residual confounding related to disease severity cannot be excluded. Second, important clinical information was unavailable in the NIS. These include underlying seizure etiology, antiseizure medication use, and other unmeasured confounders. Although patients with CNS tumors and traumatic brain injury were excluded and relevant neurologic comorbidities were adjusted for, residual confounding related to unmeasured factors remains possible. In addition, the lack of standardized inpatient pharmacy data precluded evaluation of medication-related effects, which may influence frailty and clinical outcomes. Third, due to the structure of administrative data, temporal relationships between diagnoses cannot be definitively established. As a result, some outcomes classified as “complications” may represent the primary reason for hospitalization rather than true in-hospital events, particularly for conditions such as acute myocardial infarction and cerebrovascular events. These findings should therefore be interpreted with caution. Fourth, outcome misclassification may have occurred. Specifically, “unfavorable discharge” could not reliably distinguish new institutionalization from return to a preexisting long-term care facility, and thus may partly reflect baseline residence status. Furthermore, the NIS does not provide follow-up data after discharge, precluding assessment of long-term outcomes. Fifth, the cross-sectional nature of the study limits causal inference, and the directionality of the association between frailty and epilepsy severity cannot be determined. In addition, as this study is based on hospitalized patients, the generalizability of the findings to community-dwelling individuals or those in long-term care settings remains uncertain. Finally, frailty was assessed using a single predefined index; therefore, differences in performance across alternative frailty measures could not be evaluated. Future studies comparing multiple frailty indices may help validate and extend the generalizability of our findings.

## Conclusions

Among hospitalized adults aged ≥ 60 years with epilepsy in the United States, frailty is highly prevalent, affecting approximately 58% of patients. Frailty was independently associated with adverse in-hospital outcomes, including higher risks of urinary tract infection, prolonged length of stay, and unfavorable discharge, as well as increased healthcare resource utilization.

Frailty was associated with a lower risk of in-hospital mortality, likely reflecting differences in discharge patterns, as frail patients are more often transferred to post-acute care settings. These findings highlight the importance of recognizing and addressing frailty in older patients with epilepsy to improve clinical outcomes and optimize care quality.

## Supplementary Information

Supplementary Material 1.

## Data Availability

All of the data supporting underlying findings are included in the manuscript and its supplemental files.
