# Supplementary material for: Frailty and outcomes in older adults hospitalized for epilepsy: a nationwide inpatient sample study
Source: BMC Geriatr. 2026 Jun 10;26:1063. doi: 10.1186/s12877-026-07790-3 (PMC13480203; doi:10.1186/s12877-026-07790-3)
Supplement: Supplementary file 1 [file 12877_2026_7790_MOESM1_ESM.docx]

**Supplementary Table 1. ICD codes used in the study**

|  | ICD-9 | ICD-10 |
| --- | --- | --- |
| Epilepsy | **CM**: 345.00–345.9 | **CM**: G40 |
| Febrile seizure, post-traumatic seizures or other convulsions | **CM**: 780.31, 780.32, 780.33, 780.39 | **CM**: R56 |
| TBI | **CM**: 800-801, 803-804, 850-854, 950.1-950.3, 959.01 | **CM**: S02.0, S02.1, S02.8, S02.91, S04.02, S04.03, S04.04, S06, S07.1 |
| CNS tumor | **CM**: 191, 192, 225, 237.5, 237.6, 239.6, 194.3, 227.3, 227.4, 237.0, 237.1 | **CM**: C70, C71, C72, D32, D33, D42, D43, D49.6, C75.1–C75.3, D35.2–D35.4, D44.3–D44.5 |
| AMI | **CM**: 410. [0-9] 0, 410. [0-9] 1 | **CM**: I21, I22 |
| VTE | **CM**: 415, 451-453, 671, 673, 997.2 | **CM**: I260, I269, I801-803, I808, I809, I820-I823, I828, I829, O082, O223, O871, O882, I81, I82 |
| Pneumonia | **CM**: 486, 481, 482.8, 482.3 | **CM**: A48.1, J12-J18 |
| UTI | **CM**: 599.0, 996.64 | **CM**: N39.0, T83.5 |
| Sepsis/SIRS | **CM**: 038, 995.91, 995.92, 785.52, 790.7 | **CM**: A40, A41, R65.20, R65.21, R78.81, R65.10, R65.11 |
| Smoking | **CM**: 305.1, V15.82, 989.84 | **CM**: Z71.6, Z72.0, Z86.43, Z87.891, F17, O99.33, T65.2 |
| CNS infection | **CM**: 320, 321, 322, 323, 324 | **CM**: G00, G01, G02, G03, G04, G05, G06 |
| Parkinson disease | **CM**: 332.0 | **CM**: G20.A1, G20.A2, G20.B1, G20.B2 |
| Ischemic stroke | **CM**: 433. [0-9] 1, 434. [0-9] 1, 436 | **CM**: I63 |
| TIA | **CM**: 435.0, 435.1, 435.2, 435.8, 435.9, 362.34 | **CM**: G45.0, G45.1, G45.2, G45.4, G45.8, G45.9, G45.3 |
| Hemorrhagic stroke | **CM**: 430, 431, 432 | **CM**: I60, I61, I62 |
| Intractable epilepsy | **CM**: 345.01, 345.11, 345.41, 345.51, 345.81, 345.91 | **CM**: G40.A11, G40.A19, G40.311, G40.319, G40.411, G40.419, G40.211, G40.219, G40.011, G40.019, G40.111, G40.119, G40.803, G40.804, G40.813, G40.814, G40.911, G40.919 |
| Epilepsy surgery | **PCS**: 01.52, 01.53, 01.59 | **PCS**: 00T70ZZ, 00B70ZZ, 00B00ZZ |
| AIDS |  | **CM**: B20-B22, B24 |
| Alcohol abuse |  | **CM**: F10, E52, G62.1, I42.6, K29.2, K70.0, K70.3, K70.9, T51.x, Z50.2, Z71.4, Z72.1 |
| Anemia (chronic blood loss) |  | **CM**: D50.0 |
| Anemia (deficiency) |  | **CM**: D50.8, D50.9, D51-D53 |
| Rheumatoid arthritis/collagen vascular diseases |  | **CM**: L94.0, L94.1, L94.3, M05, M06, M08, M12.0, M12.3, M30, M31.0-M31.3, M32-M35, M45, M46.1, M46.8, M46.9 |
| Congestive heart failure |  | **CM**: I09.9, I11.0, I13.0, I13.2, I25.5, I42.0, I42.5-I42.9, I43, I50, P29.0 |
| Chronic pulmonary disease |  | **CM**: I27.8, I27.9, J40-J47, J60-J67, J68.4, J70.1, J70.3 |
| Coagulopathy |  | **CM**: D65-D68, D69.1, D69.3-D69.6 |
| Diabetes (uncomplicated) |  | **CM**: E10.0, E10.1, E10.9, E11.0, E11.1, E11.9, E12.0, E12.1, E12.9, E13.0, E13.1, E13.9, E14.0, E14.1, E14.9 |
| Diabetes (complicated) |  | **CM**: E10.2-E10.8, E11.2-E11.8, E12.2-E12.8, E13.2-E13.8, E14.2-E14.8 |
| Drug abuse |  | **CM**: F11-F16, F18, F19, Z71.5, Z72.2 |
| Hypertension |  | **CM**: I10, I11-I13, I15 |
| Hypothyroidism |  | **CM**: E00-E03, E89.0 |
| Liver disease |  | **CM**: B18, I85, I86.4, I98.2, K70, K71.1, K71.3-K71.5, K71.7, K72-K74, K76.0, K76.2-K76.9, Z94.4 |
| Lymphoma |  | **CM**: C81-C85, C88, C96, C90.0, C90.2 |
| Fluid/electrolyte disorders |  | **CM**: E22.2, E86, E87 |
| Metastatic cancer |  | **CM**: C77-C80 |
| Obesity |  | **CM**: E66 |
| Peripheral vascular disorders |  | **CM**: I70, I71, I73.1, I73.8, I73.9, I77.1, I79.0, I79.2, K55.1, K55.8, K55.9, Z95.8, Z95.9 |
| Pulmonary circulation disorders |  | **CM**: I26, I27, I28.0, I28.8, I28.9 |
| Renal failure |  | **CM**: N17-N19 |
| Non-metastatic cancer |  | **CM**: C00-C26, C30-C34, C37-C41, C43, C45-C58, C60-C76, C97 |
| Valvular disease |  | **CM**: A52.0, I05-I08, I09.1, I09.8, I34-I39, Q23.0-Q23.3, Z95.2-Z95.4 |

ICD, International Classification of Disease; CM, Clinical Modification; PCS, Procedure Code System; TBI, traumatic brain injury; CNS, central nervous system; AMI, acute myocardial infarction; VTE, venous thromboembolism; UTI, urinary tract infection; SIRS, systemic inflammatory response syndrome; TIA, transient ischemic attack; AIDS, acquired immunodeficiency syndrome.

**Supplementary Table 2. ICD codes of the ACG frailty indicator**

|  | ICD-9 | ICD-10 |
| --- | --- | --- |
| Dementia and Delirium | **CM**: 290.4, 294.1-294.2, 293.0-293.1, V40.31, 331.0, 331.2, 290.0-290.3, 331.19, 331.82, 290.8-290.9, 294.8-294.9, 294.0 | **CM**: F00, F01, F02, F03, F05, G30, G31.1, G31.0, F04, R41 |
| Mobility Problems | **CM**: 781.2, 781.99 | **CM**: R26, R298 |
| Falls and Fractures | **CM**: 805.4-805.5, 806.4-806.5, 805.6-805.7, 806.6-806.7, 808-809, 839.2-839.3, 839.41-839.52, 846, 847.2-847.4, 848.5, 810-812, 831-835, 840-843, 839.61, 839.71, 848.4, 813-817, 820-821, E88, 733.0, 733.1, V15.88, 780.2, 797, 996.4 | **CM**: S32, S33, S42, S43, S52, S53, S62, S63, S72, S73, W0-W1, M80, M81, R29.6, R55, R54, M96.6 |
| Pressure Ulcers and Weight Loss | **CM**: 707.2, 707.0, 783.2, V69.1 | **CM**: L89, R63.4, R63.6, Z72.4 |
| Incontinence | **CM**: 788.3, 787.6 | **CM**: R32, R15 |
| Dependence and Care | **CM**: V60.4, V63 | **CM**: Z74, Z75 |
| Anxiety and Depression | **CM**: 296.9, 300.0, 308, 309, 300.1, 296.2, 296.3, 296.5, 300.4, 309.0, 309.1, 309.2, 2968, 298.0, 301.1, 311 | **CM**: F38, F41, F43, F44, F06.4, F32, F33, F20.4, F25.1, F31, F34.1, F41.2, F43.2 |

ICD, International Classification of Disease; CM, Clinical Modification.

**Supplementary Table 3. Characteristics of epilepsy patients categorized by frailty status before PSM**

|  |  | Frailty | |  |
| --- | --- | --- | --- | --- |
|  | All patients  (n = 176,884) | Frail  (n = 102,040) | Non-frail  (n = 74,844) | SMD |
| **Demography** |  |  |  |  |
| **Age, years** | 72.4 ± 0.03 | 73.5 ± 0.04 | 70.8 ± 0.04 | **0.115** |
| 60-69 | 79284 (44.9) | 40350 (39.6) | 38934 (52.0) | **0.303** |
| 70-79 | 55012 (31.1) | 32201 (31.6) | 22811 (30.5) |  |
| >= 80 | 42588 (24.0) | 29489 (28.8) | 13099 (17.5) |  |
| **Sex** |  |  |  | **0.230** |
| Male | 83065 (47.0) | 42989 (42.2) | 40076 (53.6) |  |
| Female | 93819 (53.0) | 59051 (57.8) | 34768 (46.4) |  |
| **Race/ethnicity** |  |  |  | **0.122** |
| White | 105621 (59.8) | 63429 (62.2) | 42192 (56.4) |  |
| Black | 39098 (22.2) | 20940 (20.6) | 18158 (24.3) |  |
| Hispanic | 13457 (7.6) | 7627 (7.5) | 5830 (7.8) |  |
| Other/unknown | 18708 (10.5) | 10044 (9.7) | 8664 (11.4) |  |
| **Insurance status / primary payer** | |  |  | **0.170** |
| Medicare/Medicaid | 150030 (84.9) | 89217 (87.5) | 60813 (81.4) |  |
| Private including HMO | 19841 (11.2) | 9475 (9.3) | 10366 (13.9) |  |
| Self-pay/no-charge/other | 6770 (3.8) | 3222 (3.2) | 3548 (4.8) |  |
| Missing | 243 | 126 | 117 |  |
| **Household income** | |  |  | 0.052 |
| Q1 | 55961 (32.4) | 31394 (31.4) | 24567 (33.7) |  |
| Q2 | 43172 (24.9) | 24933 (24.9) | 18239 (24.9) |  |
| Q3 | 39328 (22.7) | 23302 (23.2) | 16026 (21.9) |  |
| Q4 | 34806 (20.1) | 20485 (20.5) | 14321 (19.6) |  |
| Missing | 3617 | 1926 | 1691 |  |
| **Smoking** | 55200 (31.2) | 31394 (30.8) | 23806 (31.8) | 0.022 |
| **CNS infection** | 865 (0.5) | 473 (0.5) | 392 (0.5) | 0.008 |
| **Parkinson disease** | 2361 (1.3) | 1581 (1.5) | 780 (1.0) | 0.044 |
| **Ischemic stroke** | 3460 (2.0) | 1934 (1.9) | 1526 (2.0) | 0.010 |
| **TIA** | 2306 (1.3) | 1280 (1.3) | 1026 (1.4) | 0.010 |
| **Hemorrhagic stroke** | 2209 (1.3) | 1197 (1.2) | 1012 (1.4) | 0.016 |
| **Intractable epilepsy** | 9755 (5.5) | 5568 (5.5) | 4187 (5.6) | 0.006 |
| **Epilepsy surgery** | 242 (0.1) | 96 (0.1) | 146 (0.2) | 0.026 |
| **Elixhauser Comorbidities** | |  |  |  |
| AIDS | 402 (0.2) | 228 (0.2) | 174 (0.2) | 0.002 |
| Alcohol abuse | 15066 (8.6) | 7936 (7.8) | 7130 (9.6) | 0.063 |
| Anemia (chronic blood loss) | 525 (0.3) | 316 (0.3) | 209 (0.3) | 0.006 |
| Anemia (deficiency) | 18954 (10.6) | 11390 (11.1) | 7564 (10.0) | 0.034 |
| Rheumatoid arthritis/collagen vascular diseases | 4494 (2.5) | 2880 (2.8) | 1614 (2.2) | 0.043 |
| Congestive heart failure | 24795 (14.1) | 14653 (14.4) | 10142 (13.6) | 0.024 |
| Chronic pulmonary disease | 33793 (19.1) | 20548 (20.1) | 13245 (17.7) | 0.062 |
| Coagulopathy | 4967 (2.8) | 2659 (2.6) | 2308 (3.1) | 0.029 |
| Diabetes (uncomplicated) | 34237 (19.3) | 18843 (18.4) | 15394 (20.5) | 0.052 |
| Diabetes (complicated) | 19697 (11.2) | 11797 (11.6) | 7900 (10.6) | 0.032 |
| Drug abuse | 7289 (4.1) | 4272 (4.2) | 3017 (4.1) | 0.007 |
| Hypertension | 130175 (73.7) | 76504 (75.0) | 53671 (71.8) | 0.074 |
| Hypothyroidism | 29723 (16.8) | 19379 (19.0) | 10344 (13.8) | **0.140** |
| Liver disease | 5982 (3.4) | 3284 (3.2) | 2698 (3.6) | 0.021 |
| Lymphoma | 1393 (0.8) | 684 (0.7) | 709 (0.9) | 0.031 |
| Fluid/electrolyte disorders | 64561 (36.6) | 38897 (38.2) | 25664 (34.4) | 0.079 |
| Metastatic cancer | 5240 (3.0) | 2168 (2.1) | 3072 (4.1) | **0.115** |
| Obesity | 13082 (7.4) | 7612 (7.5) | 5470 (7.3) | 0.006 |
| Peripheral vascular disorders | 10937 (6.2) | 6545 (6.4) | 4392 (5.9) | 0.023 |
| Pulmonary circulation disorders | 3836 (2.2) | 2241 (2.2) | 1595 (2.1) | 0.005 |
| Renal failure | 35563 (20.2) | 20922 (20.6) | 14641 (19.7) | 0.023 |
| Non-metastatic cancer | 5212 (3.0) | 2567 (2.5) | 2645 (3.6) | 0.060 |
| Valvular disease | 9866 (5.6) | 5881 (5.8) | 3985 (5.3) | 0.020 |
| **Elixhauser comorbidity score** | |  |  | **0.102** |
| 0 | 12299 (6.9) | 6105 (6.0) | 6194 (8.2) |  |
| 1 | 32029 (18.1) | 17940 (17.5) | 14089 (18.8) |  |
| 2 | 43015 (24.3) | 24915 (24.4) | 18100 (24.2) |  |
| 3+ | 89541 (50.7) | 53080 (52.1) | 36461 (48.8) |  |
| **Year of admission** |  |  |  | **0.174** |
| 2005-2009 | 28180 (15.5) | 14251 (13.6) | 13929 (18.1) |  |
| 2010-2014 | 51298 (28.8) | 28112 (27.3) | 23186 (30.8) |  |
| 2015-2018 | 47452 (27.1) | 28459 (28.2) | 18993 (25.7) |  |
| 2019-2022 | 49954 (28.6) | 31218 (30.9) | 18736 (25.4) |  |
| **Emergent admission** | 165886 (93.9) | 96273 (94.5) | 69613 (93.2) | 0.057 |
| Missing | 311 | 196 | 115 |  |
| **Weekend admission** | 46022 (26.0) | 26662 (26.1) | 19360 (25.9) | 0.006 |
| **Hospital bed size** |  |  |  | 0.051 |
| Small | 27340 (15.4) | 16470 (16.1) | 10870 (14.5) |  |
| Medium | 48985 (27.8) | 28520 (28.1) | 20465 (27.5) |  |
| Large | 100174 (56.8) | 56861 (55.9) | 43313 (58.0) |  |
| Missing | 385 | 189 | 196 |  |
| **Hospital region** |  |  |  | 0.039 |
| Northeast | 40148 (22.7) | 22922 (22.5) | 17226 (23.1) |  |
| Midwest | 37183 (21.0) | 22069 (21.6) | 15114 (20.2) |  |
| South | 70286 (39.8) | 40495 (39.7) | 29791 (39.8) |  |
| West | 29267 (16.5) | 16554 (16.2) | 12713 (16.9) |  |
| **Hospital location/teaching status** | |  |  | 0.015 |
| Rural | 14720 (8.3) | 8423 (8.3) | 6297 (8.4) |  |
| Urban nonteaching | 50357 (28.4) | 28797 (28.2) | 21560 (28.7) |  |
| Urban teaching | 111422 (63.3) | 64631 (63.6) | 46791 (62.9) |  |
| Missing | 385 | 189 | 196 |  |

Abbreviations: HMO, health maintenance organization; CNS, central nervous system; TIA, transient ischemic attack; AIDS, acquired immunodeficiency syndrome; PSM, propensity score matching; SMD, standardized mean difference.

Continuous variables are presented as mean ± SE; categorical variables are presented as unweighted counts (weighted percentage).

SMDs ≥ 0.1 are shown in bold.

**Supplementary Table 4. In-hospital outcomes of epilepsy patients categorized by frailty status before PSM**

|  |  | Frailty | |  |
| --- | --- | --- | --- | --- |
|  | All patients  (n = 176,884) | Frail  (n = 102,040) | Non-frail  (n = 74,844) | p-value |
| **In-hospital outcomes** | |  |  |  |
| **Major complication** | 40959 (23.2) | 25812 (25.3) | 15147 (20.3) | **<0.001** |
| AMI | 2296 (1.3) | 1404 (1.4) | 892 (1.2) | **<0.001** |
| Cardiogenic shock | 208 (0.1) | 107 (0.1) | 101 (0.1) | 0.072 |
| VTE | 3466 (2.0) | 1976 (1.9) | 1490 (2.0) | 0.428 |
| Pneumonia | 7608 (4.3) | 4411 (4.3) | 3197 (4.3) | 0.632 |
| UTI | 28292 (16.0) | 18544 (18.1) | 9748 (13.0) | **<0.001** |
| Sepsis/SIRS | 5590 (3.2) | 3256 (3.2) | 2334 (3.1) | 0.406 |
| **Prolonged LOS, day ^a, b^** | 45543 (26.3) | 28893 (28.9) | 16650 (22.7) | **<0.001** |
| **Unfavorable discharge ^a^** | 57834 (33.3) | 39938 (39.8) | 17896 (24.4) | **<0.001** |
| **Total hospital cost, thousand USD** | 48.0 ± 0.3 | 49.3 ± 0.4 | 46.3 ± 0.4 | **<0.001** |
| **In-hospital mortality** | 3335 (1.9) | 1811 (1.8) | 1524 (2.0) | **<0.001** |

Abbreviations: AMI, acute myocardial infarction; VTE, venous thromboembolism; UTI, urinary tract infection; SIRS, systemic inflammatory response syndrome; LOS, length of hospital stay; PSM, propensity score matching.

Continuous variables are presented as mean ± SE; categorical variables are presented as unweighted counts (weighted percentage).

^a^ Excluding patients who died in the hospital.

^b^ Length of hospital stay ≥ 75^th^ percentile is 6 days.

p-values < 0.05 shown in bold.
